# BMC Surgery reviewer acknowledgement, 2015

**DOI:** 10.1186/s12893-016-0121-x

**Published:** 2016-02-05

**Authors:** Guangde Tu

**Affiliations:** Room 1504-1505 Financial Plaza, No. 333 Jiujiang Road, Huangpu District Shanghai, 200001 China

## Abstract

The editors of BMC Surgery would like to thank all our reviewers who have contributed their time to the journal in Volume 15 (2015).

**Thad Abrams**

USA

**Ahmed Abu-Zaid**

Saudi Arabia

**Bas Aerts**

Netherlands

**Cheguevara Afaneh**

USA

**Ayman Agha**

Germany

**Riaz Agha**

United Kingdom

**Ferdinando Agresta**

Italy

**Tolga Aksu**

Turkey

**Jason Akulian**

USA

**Ali Al Kaissi**

Austria

**Sami Alasari**

Republic of Korea

**Olusegun Alatise**

Nigeria

**Luca Antonio Aldrighetti**

Italy

**Mario Alessiani**

Italy

**Marco Allaix**

Italy

**Fernando Andrés Alvarez**

Argentina

**Emad Aly**

United Kingdom

**Rahul Anand**

USA

**Roland Andersson**

Sweden

**Kristoffer Andresen**

Denmark

**Hagen Andruszkow**

Germany

**Andrea Angelini**

Italy

**Stefano Angioni**

Italy

**Amedeo Anselmi**

France

**Stavros Antoniou**

Germany

**Luca Arru**

Luxembourg

**Nuhi Arslani**

Slovenia

**Avo Artinyan**

USA

**Osman Asıcıoglu**

Turkey

**Javier Ata-Ali**

Spain

**Sam Atallah**

USA

**Szijarto Attila**

Hungary

**Egemen Ayhan**

Turkey

**Berk Aykut**

Germany

**Berk Aykut**

Germany

**Dan Azagury**

USA

**Negar Azarpira**

Iran

**Seung Hyuk Baik**

Republic of Korea

**Nicolaas A Bakker**

Netherlands

**Andreas Bakoyiannis**

Greece

**Ugur Bal**

Turkey

**Chad Ball**

Canada

**Gordon Bannister**

United Kingdom

**Vickie Baracos**

Canada

**Louise Barbier**

France

**Sorin Traian Barbu**

Romania

**Riyaz Bashir**

USA

**Giacomo Batignani**

Italy

**Hasan Batirel**

Turkey

**Dirk Bausch**

Germany

**Kemal Behzatoglu**

Turkeuy

**Guido Beldi**

Switzerland

**Christophe Berney**

Australia

**Emilio Bertani**

Italy

**Samer Saad Bessa**

Egypt

**Roland Biber**

Germany

**Christoph Birkenmaier**

United Kingdom

**Christopher Bliemel**

Germany

**Joerg Boehme**

Germany

**Marja Boermeester**

Netherlands

**Ugo Boggi**

Italy

**Therezia Bokor**

Germany

**Martin Bolli**

Switzerland

**Elfriede Bollschweiler**

Germany

**Luigi Bonavina**

Italy

**Giuseppe Borzellino**

Italy

**Sotirios Botaitis**

Greece

**Florin Botea**

Romania

**Ismail Bouhout**

Canada

**Mehmet Abdussamet Bozkurt**

Turkey

**Federico Bozzetti**

Italy

**Stefan Breitenstein**

Switzerland

**Guy Brock**

USA

**Kristoffer W. Brudvik**

Norway

**Jacek Budzynski**

Poland

**Laura Buggio**

Italy

**Orhan Bulut**

Denmark

**Matthew Burg**

USA

**Mario Cabraja**

Germany

**Ufuk Cagirici**

Turkey

**Pietro Giorgio CALO**

Italy

**Fabio Cesare Campanile**

Italy

**Silvestro Canonico**

Italy

**Cédric Carrié**

France

**Abigail Caudle**

USA

**Giuseppe Cavallaro**

Italy

**Arif Burak Cekic**

Turkey

**Muazez Cevik**

Turkey

**Hoi-Hung Chan**

Taiwan

**Nikhil Chanani**

USA

**Edward Chang**

USA

**Hyun Chang**

Republic of Korea

**Hak Chang**

Republic of Korea

**Chan Yoon Cheah**

Australia

**Gregory Cherr**

USA

**Seungho Choi**

Republic of Korea

**Yoon Young Choi**

Republic of Korea

**Silky Chotai**

Republic of Korea

**Tam-Lin Chow**

Hong Kong

**Benjamin Chung**

USA

**Chun Kee Chung**

Republic of Korea

**Andrea Ciarrocchi**

Italy

**Yasemin Benderli Cihan**

Turkey

**Roberto Cirocchi**

Italy

**Matteo Commessatti**

Italy

**Giovanni Conzo**

Italy

**Daniele Coraci**

Italy

**Jose Costa-Maia**

Portugal

**Hsu Chiao-Po**

Taiwan

**Alessandro Crestani**

Italy

**Derly Cuellar**

USA

**Steven Cunningham**

USA

**Adrien Daigeler**

Germany

**Luca Dalla Paola**

Italy

**Maurits De Brauw**

Netherlands

**Maurizio De Palma**

Italy

**Martin de Santibañes**

Argentina

**Marco Del Chiaro**

Sweden

**Gianmattia del Genio**

Italy

**Aslan Demir**

Turkey

**Salil Deo**

USA

**Jacopo Desiderio**

Italy

**Salomone Di Saverio**

Italy

**Attilio Di Spiezio Sardo**

Italy

**Michael Diamond**

USA

**Johannes W. Dietrich**

Germany

**Ali Djalilian**

USA

**Bogdan Dorobantu**

Romania

**Cornelia Dotzenrath**

Germany

**Richard Douard**

France

**Michael Douek**

United Kingdom

**Traian Dumitrascu**

Romania

**Soumitra Eachempati**

USA

**Eleni Efremidou**

Greece

**Raimund Erbel**

Germany

**Francisco Escribano-Sotos**

Spain

**Christian Ewald**

Germany

**Rene Fahrner**

Germany

**Ibrahim Fakhr**

Egypt

**Wen-Liang Fang**

Taiwan

**Jodok Matthias Fink**

Germany

**Marco Fisichella**

USA

**Peter Fu**

Taiwan

**Shunjun Fu**

China

**Ryoji Fukushima**

Japan

**Patrick Gahr**

Germany

**Sivakumar Gananadha**

Australia

**Gurpreet Gandhoke**

USA

**Sandrö Gerli**

Italy

**Clemens Gerstenkorn**

Germany

**Javier Gil**

Spain

**Francesco Givigliano**

Italy

**Erkan Gökçe**

Turkey

**Allan Gottschalk**

USA

**Damien Grinsell**

Australia

**Christian Grommes**

USA

**Sebastien Guigard**

France

**Ali Guner**

Turkey

**Bulent Gunlusoy**

Turkey

**Shigong Guo**

United Kingdom

**Subhash Gupta**

India

**Paul Gurbel**

USA

**Samir Haddad**

Saudi Arabia

**Dingjun Hao**

China

**Jiandong Hao**

USA

**Sheng-Po Hao**

Taiwan

**Robert Hennings**

Germany

**Mitchell Henry**

USA

**Pierre Hepp**

Germany

**Fernando Herbella**

Brazil

**Benoit Herbert**

USA

**Michael Hermann**

Austria

**Daniel Hernández-Vaquero**

Spain

**Markus Hess**

Germany

**Andrew Hill**

New Zealand

**Martin Hoffmann**

Germany

**Alexander Hofmann**

Germany

**Joerg Holstein**

Germany

**Philipp Holzner**

Germany

**Hisanaga Horie**

Japan

**Philip Hormbrey**

United Kingdom

**Allard J. F. Hosman**

Netherlands

**Saeid Hosseini**

Iran

**Chi-Hsun Hsieh**

Taiwan

**Ching-Hua Hsieh**

Taiwan

**Yingqi Hua**

China

**Chang-Ming Huang**

China

**Hongzhang Huang**

China

**Line Hupfeld**

Denmark

**Igors Iesalnieks**

Germany

**Eva Intagliata**

Italy

**Francesco Inzirillo**

Italy

**Kimieru Ito**

Japan

**Eyal Itshayek**

Israel

**Domenico Rosario Osario Iusco**

Italy

**Hrvoje Ivekovic**

Croatia

**Tal Jacobson**

Australia

**Sunil Jagtap**

India

**Thorsten Jentzsch**

Switzerland

**Joon Jeong**

Republic of Korea

**Kevin Jones**

USA

**George Jour**

USA

**Ali Kabir**

Iran

**Erdinc Kamer**

Turkey

**Guenter Kampf**

Germany

**Hyun Kang**

Republic of Korea

**Matthias Kapischke**

Germany

**Mustafa Kaplanoglu**

Turkey

**Takahiro Karasaki**

Japan

**Manish Kasliwal**

USA

**Dezso Katalin**

Hungary

**Tatsuki Kataoka**

Japan

**Iraklis Katsoulis**

Greece

**Naresh Kaul**

Oman

**Masahiko Kawaguchi**

Japan

**Khandkar Ali Kawsar**

United Kingdom

**Oguz Kayiran**

Turkey

**Tobias Keck**

Germany

**Michael Kelly**

Australia

**Michael Kelly**

Australia

**Andrii Khomiak**

Ukraine

**Alireza Khoshnevisan**

Iran

**Ji Wan Kim**

Republic of Korea

**Wook Kim**

Republic of Korea

**Bong-Wan Kim**

Republic of Korea

**Heidrun Kjellberg**

Sweden

**Angelos Kolias**

United Kingdom

**Attila Kollàr**

Switzerland

**Kostiantyn Kopchak**

Ukraine

**Linetta Koppert**

Netherlands

**Peter-Martin Krarup**

Denmark

**Avdyl Krasniqi**

Kosovo

**Lukas Kreienbühl**

Switzerland

**Ziad Kronfol**

Qatar

**Naoshi Kubo**

Japan

**Simon Kuesters**

Germany

**David Kurland**

USA

**Andrea Kwa**

Singapore

**Gerold Labek**

Austria

**Sean Langenfeld**

USA

**Hryhoriy Lapshyn**

Germany

**Indu Lata**

India

**Weichen Lee**

Taiwan

**I. Michael Leitman**

USA

**Krzysztof Letachowicz**

Poland

**Chuang-Chi Liaw**

Taiwan

**Mario Lima**

Italy

**John Lipham**

USA

**Jeffrey Lisiecki**

USA

**Andrew Liss**

USA

**Charles Loh**

United Kingdom

**Heinz Lohrer**

Germany

**Kerstin Lorenz**

USA

**Laura Lorenzon**

Italy

**Balazs Lorincz**

Germany

**Tatiana Lubenets**

Ukraine

**Vladimir Lyadov**

Russian Federation

**Léon Maggiori**

France

**Masatoshi Makuuchi**

Japan

**Christopher Mantyh**

USA

**Glen Manzano**

USA

**Alessandra Marano**

Italy

**Luigi Marano**

Italy

**Lukas Marti**

Switzerland

**Brook Martin**

USA

**Alfonso Martínez-Nova**

Spain

**Ippei Matsumoto**

Japan

**Sarah McLaughlin**

USA

**Aziz Merchant**

USA

**Juerg Metzger**

Switzerland

**Arkadiusz Miernik**

Germany

**Eric Mirallié**

France

**Yevgen Miroshnychenko**

Ukraine

**Shimpei Miyamoto**

Japan

**Nor Azlin Mohamed Ismail**

Malaysia

**Michele Molinari**

Canada

**Edward Monaco**

USA

**Hideo Morioka**

Japan

**Sei-ichiro Motegi**

Japan

**Kanchan Mukherjee**

India

**Ashok Muniappan**

USA

**Matthew Mutch**

USA

**Ahmed Nada**

Egypt

**Satoshi Nagano**

Japan

**Toru Nagao**

Japan

**Satish Nair**

India

**Joseph Naoum**

Lebanon

**Narendra Nathoo**

USA

**Hakan Nazik**

Turkey

**Giuseppe Nigri**

Italy

**Hiroaki Niitsu**

Japan

**Bengt Novik**

Sweden

**Rumana Omar**

United Kingdom

**Tomofumi Osako**

Japan

**Sylvia Otto**

Germany

**Yaşar Ozdenkaya**

Turkey

**Alessandro Palladino**

Italy

**Stefano Palmucci**

Italy

**Nikolaos Pandis**

Switzerland

**Vani Parmar**

India

**Cem Kaan Parsak**

Turkey

**Andrea Patrizi**

France

**Gianluca Pellino**

Italy

**Jean Marc Perarnau**

France

**Mate Petricevic**

Croatia

**Niccolo Petrucciani**

Italy

**Alexander Petter-Puchner**

Austria

**Andrea Polistena**

Italy

**Constantinus Politis**

Belgium

**Hans-Christian Pommergaard**

Denmark

**Daniele Poole**

Italy

**Peter Prodinger**

United Kingdom

**Guixing Qiu**

China

**Borja Quiroga**

Spain

**Muthukumaran Rangarajan**

India

**Mohammad-Reza Rasouli**

Iran

**Vidya Rattan**

India

**Stefano Rausei**

Italy

**Geoffrey Roberts**

United Kingdom

**Eric Roeland**

USA

**Ulrich Ronellenfitsch**

Germany

**Robert Roses**

USA

**Giuseppe Rossi**

United Kingdom

**Andreas Arendtsen Rostved**

Denmark

**John Scott Roth**

USA

**Antonino Rubino**

Italy

**Shi Rui**

China

**Giorgio Ivan Russo**

Italy

**Muhammad Saaiq**

Pakistan

**Aly Saber**

Egypt

**Carlo Saccardi**

Italy

**Babak Saedi**

Iran

**Rashmi Salhotra**

India

**Ozgur Samancilar**

Turkey

**Gabriel Sandblom**

Sweden

**Virender Sangwan**

India

**Massimo Sartelli**

Italy

**Baris Saylam**

Turkey

**Oliver Schindler**

United Kingdom

**Anja Schmitt**

Switzerland

**John Scolaro**

USA

**Jonathan Sembrano**

USA

**Raffaele Serra**

Italy

**Drazen Servis**

Germany

**Yasuyuki Seto**

Japan

**Bakiah Shaharuddin**

United Kingdom

**Samad Shams Yahdati**

Iran

**Mayur Sharma**

USA

**Manish Sharma**

India

**Aali Sheen**

United Kingdom

**Ehab Shiban**

Germany

**Gianfranco Silecchia**

Italy

**Roger K.J. Simmermacher**

Netherlands

**Shashideep Singhal**

USA

**Rajeev Sinha**

India

**Anatolii Skums**

Ukraine

**Mikael Sodergren**

United Kingdom

**Mario Solej**

Italy

**Kyo Young Song**

Republic of Korea

**Carlos Souza**

Brazil

**Sanket Srinivasa**

New Zealand

**Daniel C Steinemann**

Switzerland

**Antonio Sterpetti**

Italy

**Arne Streitbuerger**

Germany

**Oliver Strobel**

Germany

**Jonas Strömberg**

Sweden

**Juan Manuel Suarez Grau**

Spain

**Francis Sutherland**

Canada

**Shugo Suzuki**

Japan

**Takahisa Suzuki**

Japan

**Dimitrios Symeonidis**

Greece

**Tomasz Szewczyk**

Poland

**Masahiro Takada**

Japan

**Eiji Tanaka**

Japan

**Judith Tanner**

United Kingdom

**Ozgur Tanriverdi**

Turkey

**Francesco Tartaglia**

Italy

**Hirotaka Tashiro**

Japan

**Mikhail Tavobilov**

Russian Federation

**Talar Tejirian**

USA

**Mallika Tewari**

India

**Jan Theopold**

Germany

**Fuzhou Tian**

China

**Eric Tjwa**

Netherlands

**Salvatore Tolone**

Italy

**Valeria Tonini**

Italy

**Ana Torres**

Spain

**Ümran Toru**

Turkey

**Mark Trombetta**

USA

**Stefan Tudor**

Romania

**Togas Tulandi**

Canada

**Akif Turna**

Turkey

**Marcel Twickler**

Belgium

**Oleksandre Tyvonchuk**

Ukraine

**Hakki Uzun**

Turkey

**Antonio Valezi**

Brazil

**Richard van Hillegersberg**

Netherlands

**Jos Jam van Raay**

Netherlands

**Henk van Santbrink**

Netherlands

**Aditya Vedantam**

USA

**Tomaz Velnar**

Slovenia

**Kalyana Veluvolu**

Public of Korea

**Jon Ver Halen**

USA

**Nereo Vettoretto**

Italy

**Brendan Visser**

USA

**Nikola Vladov**

Bulgaria

**Rajeev Vohra**

India

**Kajetan von Eckardstein**

Germany

**Monique Margaretha Jozefa Walenkamp**

Netherlands

**Chuanshun Wang**

China

**Daorong Wang**

China

**Jian Wang**

China

**Deshen Wang**

China

**Atsushi Watanabe**

Japan

**Hongbo Wei**

China

**Ulrich Friedrich Wellner**

Germany

**Gethin Williams**

United Kingdom

**Uwe Wittel**

Germany

**Michael Wolf**

USA

**Joyce Wong**

USA

**Chaoneng Wu**

China

**Wojciech Wysocki**

Poland

**Songfeng Xu**

China

**Yad Ram Yadav**

India

**Katsuhiko Yanaga**

Japan

**Shukun Yao**

China

**Sezgin Yilmaz**

Turkey

**Cheng-Har Yip**

Malaysia

**Naoyuki Yokoyama**

Japan

**Tarik Yonguc**

Turkey

**Toshitaka Yoshii**

Japan

**Takeshi Yuasa**

Japan

**Giovanni Zagli**

Italy

**Dirk Zajonz**

Germany

**Melody Zhifang Ni**

United Kingdom

**Katja Zirlik**

Germany

**Oleksii Zubkov**

Ukraine

